# Genomic epidemiological analysis of *Klebsiella pneumoniae* from Portuguese hospitals reveals insights into circulating antimicrobial resistance

**DOI:** 10.1038/s41598-022-17996-1

**Published:** 2022-08-13

**Authors:** Anton Spadar, Jody Phelan, Rita Elias, Ana Modesto, Cátia Caneiras, Cátia Marques, Luís Lito, Margarida Pinto, Patrícia Cavaco-Silva, Helena Ferreira, Constança Pomba, Gabriela J. Da Silva, Maria José Saavedra, José Melo-Cristino, Aida Duarte, Susana Campino, João Perdigão, Taane G. Clark

**Affiliations:** 1 Department of Infection Biology, Faculty of Infectious and Tropical Diseases, London School of Hygiene and Tropical Medicine, Keppel Street, London, WC1E 7HT UK; 2 Research Institute for Medicines (iMed.ULisboa), Faculdade de Farmácia, Universidade de Lisboa, Lisbon, Portugal; 3 Microbiology Research Laboratory of Environmental Health (EnviHealthMicro Lab), Institute of Environmental Health (ISAMB) and Institute of Preventive Medicine and Public Health (IMP&SP), Faculty of Medicine, Universidade de Lisboa, Lisbon, Portugal; 4 Faculdade de Medicina Veterinária, Universidade Lusófona de Humanidades E Tecnologias, Lisbon, Portugal; 5 Laboratório de Microbiologia, Serviço de Patologia Clínica, Centro Hospitalar Universitário Lisboa Norte, Lisbon, Portugal; 6 Laboratório de Microbiologia, Serviço de Patologia Clínica, Centro Hospitalar Universitário Lisboa Central, Lisbon, Portugal; 7 Centro de Investigação Interdisciplinar Egas Moniz, Instituto Universitário Egas Moniz, Caparica, Portugal; 8 Technophage, Lisboa, Portugal; 9 UCIBIO, Microbiology Service, Biological Sciences Department, Faculty of Pharmacy, University of Porto, Porto, Portugal; 10 Centre of Interdisciplinary Research in Animal Health (CIISA), Faculty of Veterinary Medicine, University of Lisbon, Avenida da Universidade Técnica, 1300-477 Lisboa, Portugal; 11 Faculty of Pharmacy and Center for Neurosciences and Cell Biology, University of Coimbra, Coimbra, Portugal; 12 Laboratory Medical Microbiology, Department of Veterinary Sciences, CITAB-Centre for the Research and Technology Agro-Environmental and Biological Sciences, University of Trás-Os-Montes and Alto Douro, Vila Real, Portugal; 13 Instituto de Microbiologia, Faculdade de Medicina, Universidade de Lisboa, Lisbon, Portugal; 14 Faculdade de Farmácia, Universidade de Lisboa, Lisbon, Portugal; 15 Faculty of Epidemiology and Population Health, London School of Hygiene and Tropical Medicine, London, UK

**Keywords:** Antibiotics, Antimicrobial resistance, Bacterial genomics

## Abstract

*Klebsiella pneumoniae* (Kp) bacteria are an increasing threat to public health and represent one of the most concerning pathogens involved in life-threatening infections and antimicrobial resistance (AMR). To understand the epidemiology of AMR of Kp in Portugal, we analysed whole genome sequencing, susceptibility testing and other meta data on 509 isolates collected nationwide from 16 hospitals and environmental settings between years 1980 and 2019. Predominant sequence types (STs) included ST15 (n = 161, 32%), ST147 (n = 36, 7%), ST14 (n = 26, 5%) or ST13 (n = 26, 5%), while 31% of isolates belonged to STs with fewer than 10 isolates. AMR testing revealed widespread resistance to aminoglycosides, fluoroquinolones, cephalosporins and carbapenems. The most common carbapenemase gene was *bla*_*KPC-3*_. Whilst the distribution of AMR linked plasmids appears uncorrelated with ST, their frequency has changed over time. Before year 2010, the dominant plasmid group was associated with the extended spectrum beta-lactamase gene *bla*_*CTX-M-15*_, but this group appears to have been displaced by another carrying the *bla*_*KPC-3*_ gene. Co-carriage of *bla*_*CTX-M*_ and *bla*_*KPC-3*_ was uncommon. Our results from the largest genomics study of Kp in Portugal highlight the active transmission of strains with AMR genes and provide a baseline set of variants for future resistance monitoring and epidemiological studies.

## Introduction

*Klebsiella pneumoniae* (Kp) is a common gram-negative pathogen associated with both hospital and community acquired infections^1^. During the past decades the association of Kp bacteria with antibiotic resistance has been increasingly recognized along with a variety of resistance mechanisms^2^. Production of laterally transferable genes encoding enzymes such as aminoglycoside-modifying enzymes drives high levels resistance to aminoglycoside antibiotics^3^. Resistance associated mutations in quinolone target enzymes (DNA gyrase and topoisomerase IV) are chromosomally encoded by the *gyrA, gyrB* and *parC* quinolone resistance-determining region as well as plasmid-mediated quinolone resistance genes^4,5^. Extended spectrum beta-lactamases (ESBLs) render inactive virtually all beta-lactam antibiotics (e.g., penicillins, cephalosporins, but not carbapenems). Nonetheless, of most concern is the increase in prevalence of carbapenemase producing Kp isolates^6^ which greatly limits the options for effective therapies and drives hospital outbreaks that promote further spread of antimicrobial resistance (AMR)^7,8^.

In Southern Europe OXA-48-like, VIM and KPC are the dominant carbapenemase families^9,10^. Knowledge of the carbapenemase landscape in Portugal is incomplete. However, the prevalence of carbapenem-resistant invasive Kp isolates in Portugal has increased from 3.4% in 2015 to 10.9% in 2019. During the same period the share of isolates with combined resistance to third generation cephalosporins, fluoroquinolones and aminoglycosides (26.5% in 2019) oscillated without an upward trend^6^. Several recent small epidemiological studies in Portugal have focused on carbapenemase producing Kp isolates^11–16^, and *bla*_*KPC-3*_ was the most frequently identified carbapenem resistance gene. The *bla*_*KPC-3*_ gene is most frequently located within the Tn*4401*d transposon, and IncN, IncFII, IncFIB and IncFIIA plasmid families are the main traffickers^11,13,15,17^. High flexibility of the Kp accessory genome is an additional concern because it acts as gateway for the introduction of new resistance genes into a broader set of gram-negative pathogens^2^.

Whole genome sequencing (WGS) has revolutionised the study of pathogens, not only through the characterisation of AMR associated mutations and plasmids^18,19^, but also through the determination of phylogenies and transmission events^20,21^. The Kp phylogeny normally forms clades consistent with the commonly used multi-locus sequence typing scheme (MLST), which is based on seven gene loci (*gapA, infB, mdh, pgi, phoE, rpoB* and *tonB*)^22^. Lipopolysaccharide (O-type) and Capsular Polysaccharide (K-type) serotype profiles can be informative for vaccine development, and > 130 capsular serotypes have been predicted from WGS data with KL2, or O1, O2, O3, and O5 serotypes accounting for most strains^23,24^. Alternatively, clonal groups (CGs) based on 694 core genes are sometimes characterised^25^. Strains from different sequence types (STs), O and K antigen types, and CGs can differ sharply in their virulence and propensity to be antibiotic resistant^25^. For example, ST23, ST26, ST57 and ST163 have been linked to Kp hypervirulence^26^. Unfortunately, the widely used Kp MLST scheme does not allow for a high-resolution phylogeny. The recombination and horizontal gene transfer within Kp also complicates a phylogenetic analysis^27^, even within the same STs^28^. Furthermore, a large part of the core genome exhibits very limited polymorphism, and mobile genetic elements may have relatively greater clinical relevance as exemplified by virulence and AMR factors^27^. More generally, these include integrative conjugative elements (ICE), which are a diverse group of chromosomally integrated, self-transmissible mobile genetic elements that are active in shaping the functions of bacteria and bacterial communities, including in Kp.

The current study aims to improve understanding of the genomic and AMR landscape of Kp in Portugal by analysing the largest WGS dataset to date, which consists of 509 isolates spanning a period between years 1980 and 2019. We examine the prevalence of STs, genes associated with AMR and virulence, and how the AMR profiles relate to plasmid replicon signatures of the isolates. We found that although ST15, ST14 and ST147 predominate, almost one-third of isolates came from STs considered infrequent in Portugal. We establish there are many AMR determinants, which are evolving over time, and our work provides a baseline set of variants for future monitoring and epidemiological studies in Portugal and wider Europe.

## Results

### In silico ST diversity and population structure of *K. pneumoniae* in Portugal

This study includes a total of 509 Kp isolates. Of these, 459 are clinical isolates from hospitals in the southern (n = 378), central (n = 20) and northern (n = 61) regions of Portugal (Table 1). These were compared to isolates collected from veterinary clinics (n = 41) and environmental wastewater (n = 9) from the southern region, thereby broadening insights into the incidence of Kp in Portugal. The isolates were collected between years 1980 and 2019, but the majority (n = 455, 89%) were collected between 2000 and 2019 (Table 1). Of the O antigen types, O1 was dominant with O1v1 (44%) and O1v2 (20%) followed by O2v2 (12%) and O2v1 (10%) serotypes (Table 1). Seventy-seven different STs were inferred, with the most frequent being ST15 (n = 161), ST147 (n = 36), ST13 (n = 26) and ST14 (n = 26) (Table 1). Globally significant clonal group 258 had little presence, with 4% of isolates belonging to ST11 and none to ST258 or ST512. A high proportion of isolates (31%) were from low frequency STs (each < 2.0%). Out of 2,926 possible unique ST pairs, only 0.7% differed by a single allele, whereas 22.7%, 32.8% and 25.7% differed by 4, 5 and 6 alleles, respectively (Fig. S2). O serotypes were linked strongly with STs (Fig. S3). Only 10 STs had isolates with different O serotypes. ST15 (n = 161) had 149, 9 and 3 isolates with O1v1, O1v2 and O1/O2v1 serotypes, respectively. ST13 (n = 26) had 25 and 1 isolates with O1v2 and O1v1 serotypes, respectively. ST14 (n = 26) had 24 and 2 isolates with O1v1 and O1/O2v1 serotypes, respectively. ST348 (n = 22) had 19 and 3 isolates with O1v1 and O1/O2v1 serotypes, respectively. ST11 (n = 19) had 13, 5 and 1 isolates with O2v2, O3b and O4 serotypes, respectively. There were a further five STs (34 isolates) containing isolates with different O serotypes. The predominant capsular K serotypes were KL112 (14%) and KL24 (19%), linked to the common ST15. When considering only the unique ST and K group combinations (n = 105), the most frequent K serotypes were KL30, KL10, and KL24 observed in 7, 7 and 5 STs, respectively.

**Table 1:** Baseline characteristics for the 509 *K. pneumoniae* study isolates.

Characteristic	N	%
**Region**		
Centre	20	3.9
South	428	84.1
North	61	12.0
**Collection dates**		
1980–1982	46	9.0
1990–1999	13	2.6
2000–2009	169	33.2
2010–2019	281	55.2
**Sequence type (ST)**		
ST15	161	31.6
ST147	36	7.1
ST13	26	5.1
ST14	26	5.1
ST348	22	4.3
ST307	20	3.9
ST11	19	3.7
ST231	16	3.1
ST70	16	3.1
ST45	12	2.4
Other**	155	30.5
**Inferred O serotypes**		
O1	329	64.6
O2	112	22.0
O3b	17	3.3
O4	13	2.6
O5	13	2.6
OL101	12	2.4
Unknown	10	2.0
O3/O3a	3	0.6
**Inferred K serotypes**		
KL24	96	16.7
KL112	71	14.8
KL64	35	7.1
KL62	32	6.4
KL3	26	5.6
Other	249	49.4
**Carbapenemases and carbapenem-hydrolyzing beta-lactamases**		
None	395	77.6
KPC-3	101	19.8
OXA-181	6	1.2
GES-5; KPC-3	5	1.0
OXA-48	1	0.2
GES-5	2	0.4
NDM-1	2	0.4
**Aminoglycoside resistance genotypes**		
AAC(3)-II; APH(3')-I; APH(6)-I;	154	30.3
AAC(6')-I; ANT(3'')-I; APH(3')-I; APH(6)-I;	64	12.6
APH(3')-I; APH(6)-I;	33	6.5
AAC(3)-II; ANT(3'')-I; APH(3')-I; APH(6)-I;	30	5.9
ANT(3'')-I;	26	5.1
AAC(3)-II; AAC(6')-I; ANT(3'')-I; APH(3')-I; APH(6)-I;	24	4.7
AAC(3)-II;	19	3.7
AAC(6')-I; ANT(3'')-I;	18	3.5
AAC(3)-II; AAC(6')-I; ANT(3'')-I;	10	2.0
ANT(3'')-I; APH(3')-I; APH(6)-I;	9	1.8
AAC(3)-II; ANT(3'')-I;	8	1.6
AAC(3)-II; ANT(3'')-I; APH(3')-I;	8	1.6
AAC(3)-II; APH(3')-I;	8	1.6

**Includes ST1138 with 7 isolates.

### Phylogenetic analysis

**Figure 1:**
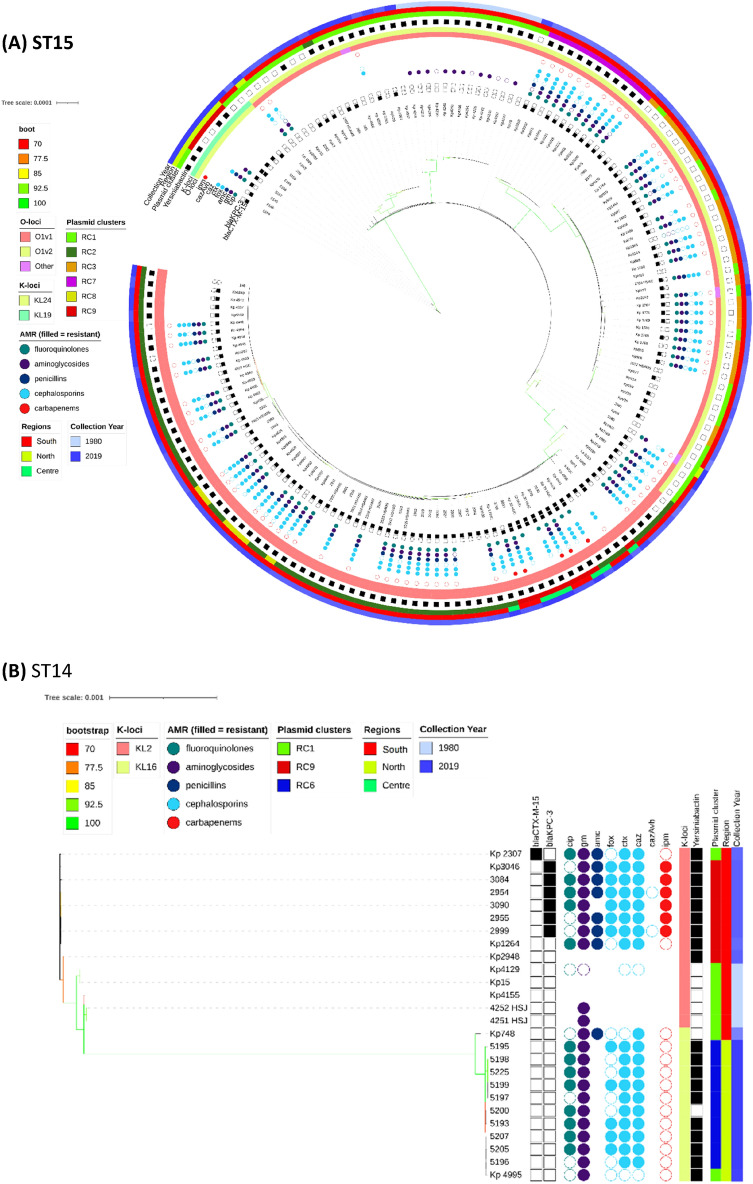

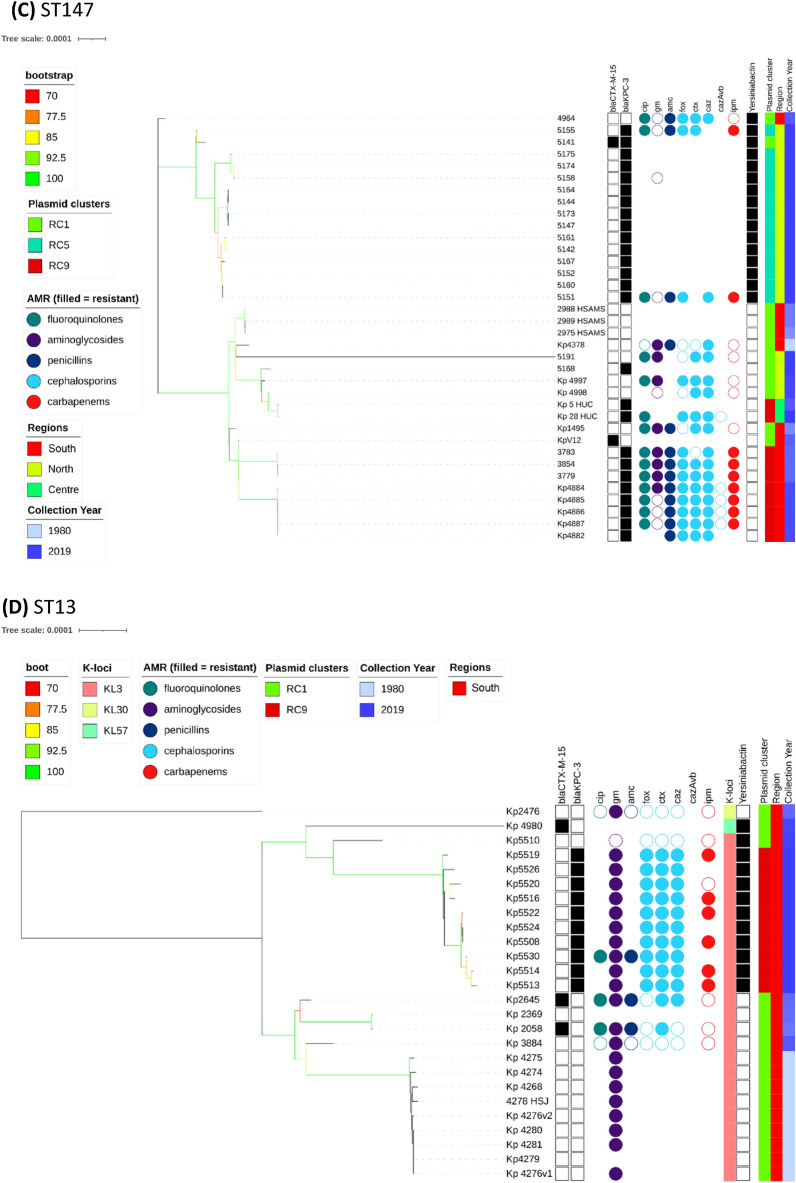
Phylogenetic trees of the most commons sequence types (STs), their antimicrobial resistance (AMR) phenotype, and carbapenemase and ESBL genotypic profiles. K-Loci refer to inferred K serotypes. Branch colours represent bootstrap support values.

**Figure 2:**
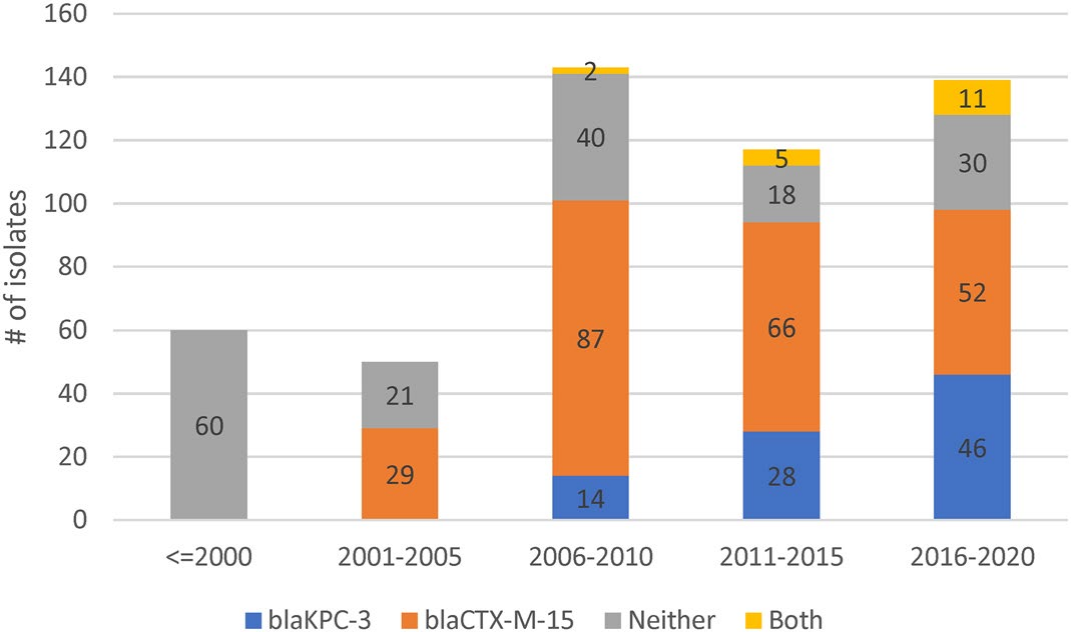
The most common ESBL (*bla*_*CTX-M-15*_) and carbapenemase (*bla*_*KPC-3*_) genes across the 509 Kp isolates by year group.

The overall phylogenetic tree (n = 509) is largely congruent with ST type, but the propensity of Kp to recombine, means that the branch support values decline rapidly as one moves from leaves to root (Fig. S3). Individual ST trees (Fig. 1A–D) identified several well supported geographic clades, including two previously reported^8,14^ potential outbreaks in Lisbon and Vila Real hospitals involving representatives of ST147 and ST15. For the most abundant sequence type ST15 (n = 161), the tree shows multiple clades differentiated by K serotypes (Fig. 1A). Nearly all isolates carried *bla*_*CTX-M-15*,_ except isolates from years 1980 to 1982 and 5 of 14 isolates from years 2007 to 2018 with *bla*_*KPC-3*_. Due to the low number of isolates spanning years 1990 to 1999, our dataset may not fully reflect the beta-lactamase diversity during that period. It has been suggested that *bla*_*TEM-10*_ was the dominant ESBL gene at that time, and most of our isolates (11/16) carried it. The smallest clade (n = 9) was from north Portugal and was distinguished by KL19 and O1v2 serotypes with five isolates carrying *bla*_*KPC-3*_. The other *bla*_*KPC-3*_ carrying ST15 isolates (n = 9) were phylogenetically very distant and part of a larger clade (n = 67) with K112 and O1v1 serotypes. Interestingly, these nine isolates carried both *bla*_*KPC-3*_ and *bla*_*CTX-M-15*_, in contrast to all other relevant isolates, which carry either *bla*_*KPC-3*_ or *bla*_*CTX- M-15*_ (Fig. 2). Four of the nine wastewater samples belonged to ST15, and were dispersed amongst clinical samples in the ST15 phylogenetic tree (Fig. 1A).

Most ST14 isolates (n = 20/26) fell within two dominant clades (Fig. 1B); the first clade (n = 11) was sourced from northern Portugal in year 2018 and contains the K16 locus, and the second (n = 9) was distinguished by the K2 serotype and spanned years 1980 to 2010. Only the second clade had *bla*_*KPC*-3_ carrying isolates (n = 6). Unlike for other STs, the genes used in phylogenetic reconstruction of ST14 were relatively concentrated within a 3.60 Mbp to 3.75 Mbp chromosomal region based on the NC_016845.1 Kp assembly (Fig. S1).

All ST147 (n = 36) isolates had KL64 and O2v1 serotypes and belonged to two main clades. One clade consisted of northern region isolates (n = 15) from year 2018, all of which carried *bla*_*KPC-3*_, but only one had *bla*_*CTX-M-15*_ (Fig. 1C). All isolates in this clade carried the siderophore yersiniabactin (*ybt 16; ICEKp12*) locus related to virulence. A second clade (n = 20) was sourced from the southern region and spanned years 1995 to 2016. Only 11 isolates in this clade carried *bla*_*KPC-3*_ and none had the yersiniabactin locus. Similarly, the ST13 tree had two clades characterised by the KL3 serotype (Fig. 1D). One clade (n = 10) was sourced from multiple hospitals in year 2019, and all isolates carried *bla*_*KPC-3*_ and yersiniabactin loci. Another clade (n = 13) spanned years 1982 to 2013 and came from the southern region. Only two isolates in this clade carried *bla*_*CTX-M-15*_, and none had other ESBL genes, *bla*_*KPC-3*_, or the yersiniabactin locus. Unlike the remaining ST13 isolates, these two phylogenetically distant isolates had inferred K57 and K30 serotypes.

Our dataset included four isolates of type ST1138, an ST which was previously described at two hospitals in Portugal^13^. We also had previously undescribed ST6003 (n = 3), which differs from ST1138 by an *rpoB* allele. All seven isolates were collected in 2012 from the same hospital in Lisbon. Remarkably, one of the ST1138 isolates has no AMR genes or mutations apart from *bla*_*SHV-36*_. This suggests its ancestors were newly introduced into the hospital system. This original strain has split into at least two lineages, both carrying *bla*_*KPC-3*_, but it is unclear if this acquisition was independent in each lineage. There are at least two inferred lineages because the isolates from overlapping dates have one of two types of *rpoB* alleles (denoted as alleles 1 or 46). We did not identify any post-2012 ST1138 isolates in our data, but it seems to have been present in Portuguese isolates collected in year 2011^13^. Apart from ST6003, our dataset had two further one allele variants of ST1138: ST514 (n = 2) collected from hospital patients in the 1980s and ST323 (n = 1) collected from wastewater in 2018. Both ST514 and ST323 came from the same geographical area as sequence types ST1138 and ST6003.

### Plasmid clustering

**Figure 3:**
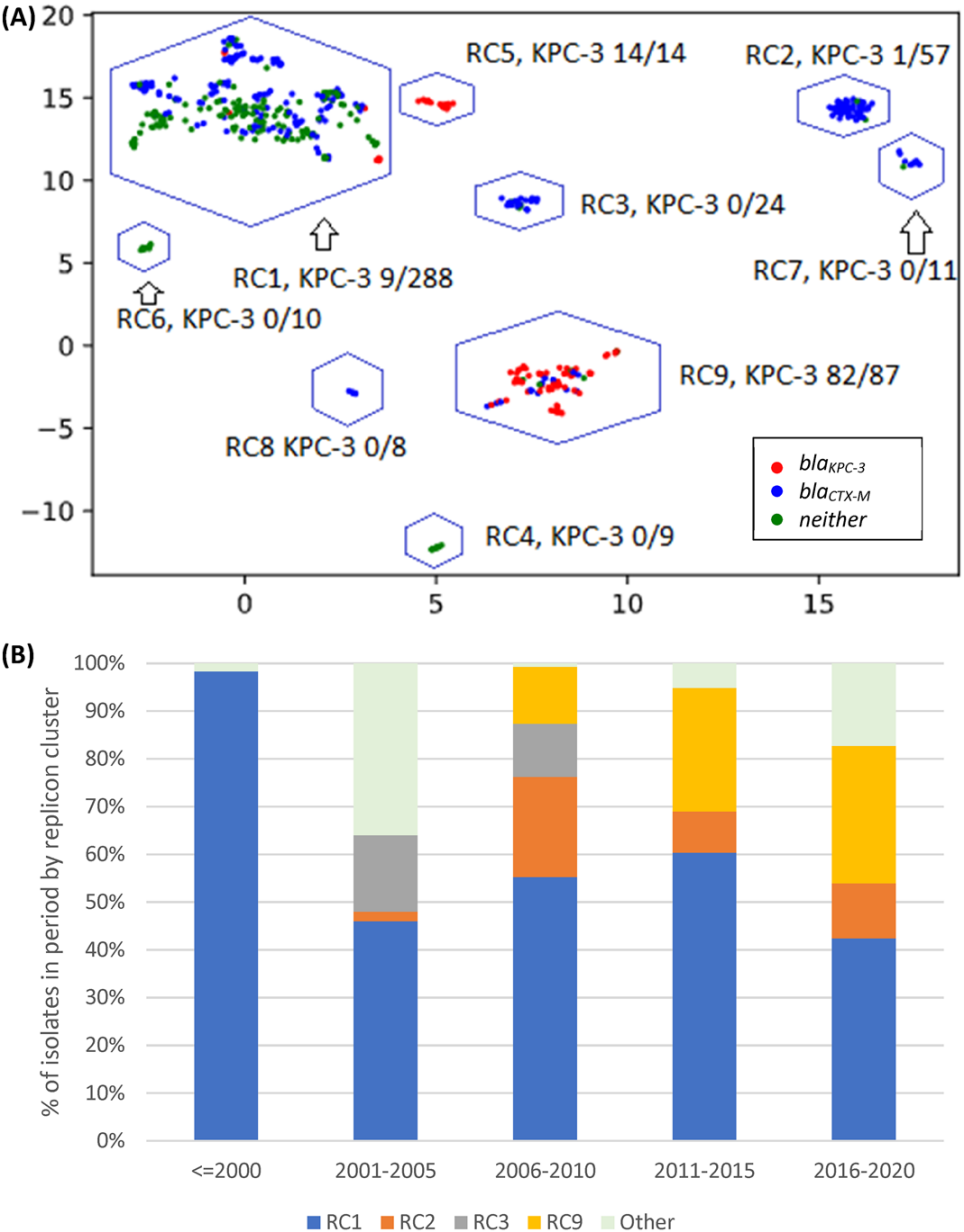
(**A**) Clustering of isolates by their plasmid replicon (replicon clusters, RC) and antimicrobial resistance (AMR) genotypic profiles, revealing differentiation by carriage of *blaKPC-3* and *blaCTX-M-15* genes. X and Y axis are dimensions on which full data is projected, they are unitless; (**B**) Abundance of isolates from different plasmid clusters.

Of the 48 replicon families identified by PlasmidFinder software, six occurred in at least 10% of isolates (IncFIB 98%, IncFII 96%, IncFIA 55%, IncR 42%. IncHI1B 11%, IncN 10%). An average of 4 replicons (range: 0–9) were identified per isolate with only three isolates having none. To understand the pattern of plasmid replicons across isolates we applied UMAP dimensional reduction methods^29^ to presence-absence matrices of replicon family (n = 48) and unique replicon sequences (n = 240). The results based on families and exact replicon sequences were consistent; so here we present the higher resolution analysis using replicon sequences (Data S1). We observed nine clear and robust clusters (replicon clusters, RCs) (Fig. 3A). RC prevalence varied over time (Fig. 3B) with RC9, the cluster with 94% *bla*_*KPC-3*_ carriage, becoming dominant between years 2010 and 2019. By overlaying RC type onto the overall and ST specific phylogenetic trees, mosaic distributions were revealed, further supporting the movement of plasmids among Kp lineages (Fig. 1A–D).

RC1 contained 57% of all isolates (n = 288), but very few (n = 9) isolates had *bla*_*KPC-3*_; although 59% carried ESBL encoding genes, which is consistent with early isolates dominating this cluster. The frequency of isolates in this cluster has declined substantially after year 2001. RC1 has a large diversity of plasmid replicons including IncFIB(K) (n = 214), IncFII(K) (n = 174), IncR (n = 108), and CoI(pHAD28) (n = 108) with each replicon family consisting of multiple distinct phylogenetic clades making RC1 interpretation complex. In contrast, the RC9 (n = 87) cluster has 25 different STs, including the dominant ST15, ST147 and ST14 types. Almost all Kp in RC9 cluster (94%, 82/87) carry *bla*_*KPC-3*_. This gene is absent in 5 isolates: ST14 from 2007 and 2010; ST416 from 2011; ST1138 from 2012; and ST359 from 2018. The key signature of RC9 is the presence of variants of FIA(pBK30683) and FII(pBK30683) replicons (Data S1). For isolates with *bla*_*KPC-3*_ in RC9, more than a third (31/87) were diverse STs collected between years 2010 and 2019, but possessing neither *gyrA* mutations, nor an *aac(6')-Ib-cr* gene. These isolates were susceptible to fluoroquinolones with average inhibition zone diameters of 20 mm among 27 tested isolates.

The RC5 cluster (n = 14) consisted of ST147 carrying *bla*_*KPC-3*_ and was congruent with the clade sourced from northern Portugal in year 2018 (n = 15). Of these northern Portuguese isolates, 14 belonged to RC5 and one belonged to RC1 (Fig. 1C). The RC5 cluster is identifiable by two variants of IncN and IncFIB(pKPHS1) replicons (Data S1). IncN is uncommon in our dataset (51/2222 replicons) and its strong link with *bla*_*KPC-3*_ suggests, in line with earlier reports^15,30^, that *bla*_*KPC-3*_ may be mobilized by IncN plasmids. In contrast, other clusters had homogenous ST types: RC2, RC3, RC7 and RC8 consist of 57, 24, 11, and 8 ST15 isolates; RC6 consists of ten ST14 isolates; and RC4 of nine ST12 isolates. RC3 is interesting because despite having isolates from years 2003 to 2014, only one (1/24) had a *bla*_*SHV*_ type beta-lactamase (*bla*_*SHV-11*_). These genes are very common (87.4%) and are normally chromosomal, which suggests either the loss of *bla*_*SHV*_ or simultaneous circulation of several strains.

All RC2 isolates carried IncFIB(K) and IncFII(pKP91) replicons, where the associated variants were different to those from the RC1 replicons. The RC2 IncFIB(K) variant was also present in RC8 and RC7 isolates. RC7 isolates also carried a distinct variant of a Col440I replicon, and a variant of IncR is shared with RC1, RC2, RC3 and RC8. RC8 carried a distinct version of IncFII(K) and IncFIB(pQil) replicons. Finally, in addition to shared IncR and ColpVC variants, RC3 also carried IncHI1A and IncHI1B(R27) replicons, which were absent in other clusters (Data S1).

### Antimicrobial resistance genotypes and phenotypes

In vitro AMR profiles and genotypes were analysed for the 509 Kp isolates, with some gaps depending on the decade of phenotypic assessment (Table S1). Most isolates underwent antibiotic susceptibility testing for aminoglycosides (n = 356, 68.1%), cephalosporins (n = 366, 71.2%), fluoroquinolones (n = 344, 66.9%), carbapenems (n = 296, 57.6%), and penicillins (n = 311, 60.5%) (Table S1). However, 121 isolates had no AMR susceptibility data. Since AMR testing was performed over multiple years, the concentration of active compounds in disks used might vary between isolates; all breakpoints were determined based on EUCAST v11.0 (2021)^31^.

### Beta-lactams

**Figure 4:**
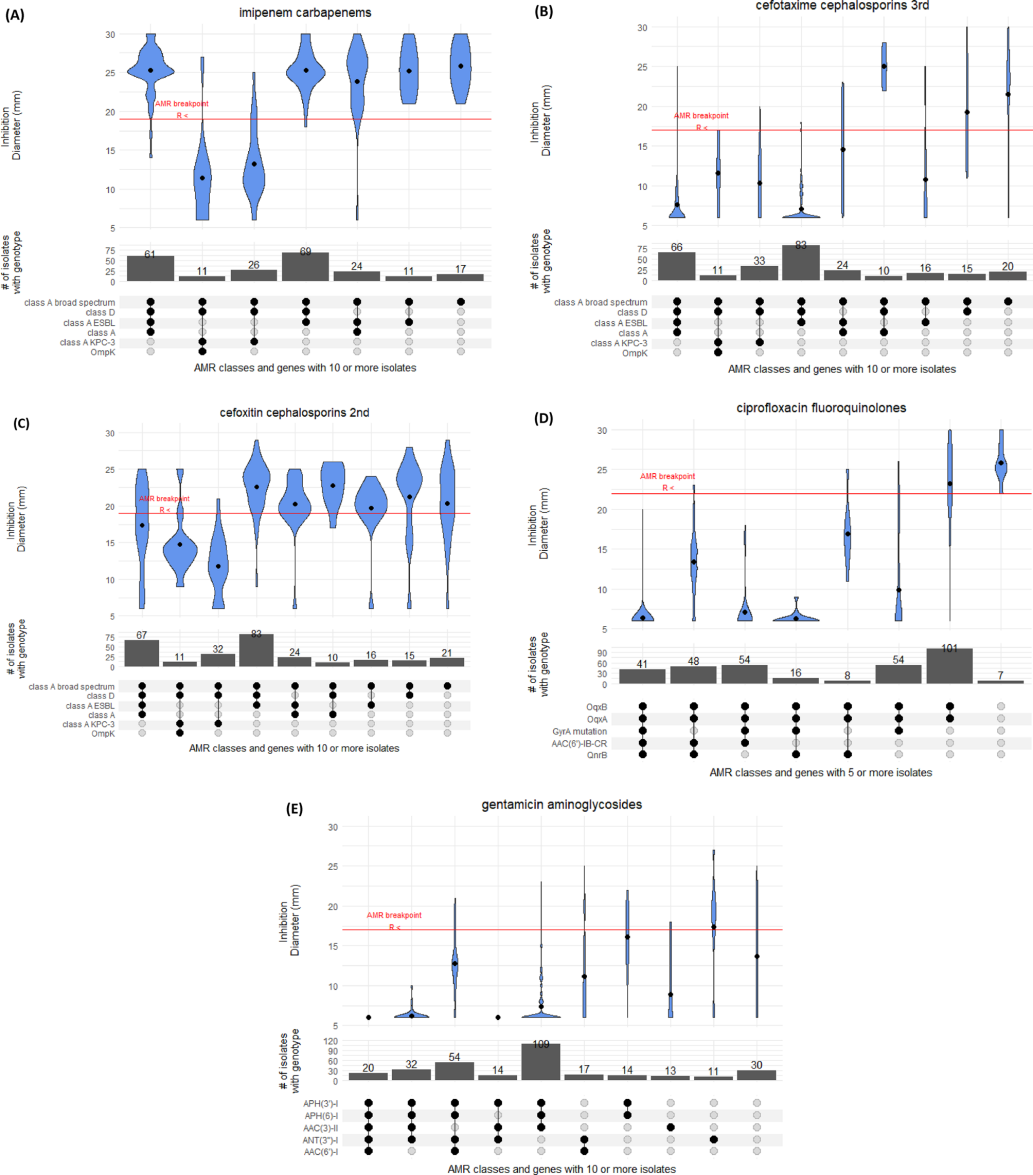
Distribution of inhibition zone diameters for different genotypes for (**A**) imipenem, (**B**) cefotaxime, (**C**) cefoxitin, (**D**) ciproflocaxin, and (**E**) gentamicin antimicrobials.

The majority (75%) of 304 tested isolates showed susceptibility to imipenem; an antibiotic used widely in hospital clinical practice in Portugal (Fig. 4). The resistance driver was likely *bla*_*KPC-3*_ (n = 106), which was carried on a Tn4401d transposon in nearly all cases (n = 101/106). In four isolates [ST15 (Kp5149), ST147 (Kp5147), ST34 (Kp5148) and ST461 (Kp5162)], the *bla*_*KPC-3*_ gene has undergone an identical inversion within the Tn4401d structure. Only one of these four isolates (ST147) was tested for imipenem resistance and was determined to be resistant (inhibition zone diameter of 6 mm). This observation suggests the inversion did not significantly impair the resistance conferred by the presence of *bla*_*KPC-3*_. Additionally, our dataset contained three isolates without carbapenemase genes but resistant to imipenem. These three isolates had no clear commonality between them, nor clear distinction from susceptible isolates with a similar genotype.

Resistance to cephalosporins was widespread with 68% of 335 tested isolates showing resistance to cefotaxime, a third-generation cephalosporin, driven likely by the presence of class A ESBL *bla*_*CTX-M-15*_ (n = 213, 41%) (Fig. 4). Ceftazidime had an even higher resistance prevalence of 91%. This drug was used widely in the 1990’s for Pseudomonas outbreaks, but is currently rarely used in monotherapies. The combination of ceftazidime and carbapenemase inhibitor avibactam (cazAvb) was rarely resistant (1/46 tests). The resistant isolate (ST15) carried *bla*_*OXA-1*_*, bla*_*TEM-1*_*, bla*_*SHV-28*_ and had truncated porin gene *ompK36*. Of the imipenem resistant isolates, twelve were tested for cazAvb and all were susceptible.

Finally, the second-generation cephalosporin cefoxitin presents an interesting case because its resistance profile was frequently inconsistent with its genomic or genotypic profile (Fig. 4). Of 366 tested isolates, 8 carried AmpC beta-lactamase *bla*_*DHA-1*_, which is associated with strong inhibition of cefoxitin. Consistent with this, these 8 isolates had a cefoxitin inhibition zone diameter of 6 mm. However, the inhibition zone diameter of the tested *bla*_*KPC-3*_ carrying isolates (n = 74) was on average 13 mm, which is below the 19 mm breakpoint, but well above the total inhibition diameter of 6 mm. In isolates with class A (non-broad-spectrum, broad-spectrum, ESBL) and D beta-lactamases, the susceptibility to cefoxitin varied vastly with isolates containing all four categories having almost uniform distribution of inhibition zone diameters between 25 and 6 mm. We were able to trace some of this inconsistency to 83 isolates with *bla*_*SHV-28*_. The mean diameter for isolates with class A broad spectrum, class A ESBL and class D genes is 22 mm. If this genotype also included *bla*_*SHV-28*_ (class A), the mean inhibition zone diameter reduced to 16 mm, but this effect is absent for other *bla*_*SHV*_ variants.

### Fluoroquinolones

Of the 307 isolates tested for ciprofloxacin susceptibility, 288 (94%) were resistant (Fig. S4). Five isolates did not have any common fluoroquinolone resistance determinants and were all susceptible with between 22 and 30 mm inhibition zone diameters. By far the strongest determinant of resistance was the presence of mutations in type II topoisomerase *gyrA. *In silico screening with Kleborate software identified simultaneous mutations GyrA83F and GyrA87A (n = 150), and single mutations GyrA83I (n = 91) and GyrA83Y (n = 15) as the most common ones. Nearly all isolates with *gyrA* mutations had inhibition zone diameters below 10 mm, whereas the resistance breakpoint is 22 mm. In 54 tested isolates that had both the *gyrA* mutation and *qnrB* gene*,* nearly all had inhibition zone diameters of 6 mm, demonstrating clear compounding of resistance. The *oqxAB* genotype gives a small decrease in susceptibility, but most of such isolates were still susceptible. Finally, *qnrB* and *qnrS* each substantially decreased inhibition zone diameter. In our dataset these genes were present only in combination with *oqxAB,* leading to reduced inhibition zone diameters of ~ 50% compared to *oqxAB* alone. As we did not have isolates with both *qnr* genes, we could not confirm if their effect is cumulative. Further, 36 and 24 isolates were tested with levofloxacin and norfloxacin, respectively, with 42% of each set showing susceptibility. In contrast to ciprofloxacin, levofloxacin was only moderately affected by *gyrA* mutations with an average inhibition zone diameter reduced to 14 mm (Fig. S4).

We examined the diversity of the *parC* gene which has been associated with fluoroquinolone resistance ^5,32^, and found 244 isolates carrying both the ParC80I mutation and *gyrA* mutations. The *parC* alleles were ST specific except for three ST307 outlying isolates that differ by a single SNP from the other seventeen ST307 and fifteen ST15 isolates (Fig. S5) which suggests a lack of selective pressure on the gene. A tree constructed using *gyrA* sequences formed clear clades for the abundant ST14 and ST15 types (Fig. S5). ST14 isolates formed two major clades, one linked to decades 1980’s and 2010’s (n = 14) and another from the 2000’s (n = 15). ST15 formed three clades, one linked to the 1980s (n = 13), and two others post-year-2000 (n = 18, n = 131). There was additional evidence of selective pressure in other STs for which we had fewer isolates (Fig. S5). We could not test the effect of *parC* mutations on resistance because all isolates with mutations in this gene also had mutations in *gyrA*. Finally, 237 isolates (46%) had an *aac(6')-Ib-cr* gene, of which 132 were tested for ciprofloxacin resistance and 128 were resistant. This gene had moderate impact on disk diameter, but this impact is sufficient for isolates to fall below resistance threshold. While the majority of *aac(6')-Ib-cr* carrying isolates also had *gyrA* mutations, we found 48 isolates with an *oqxAB*/*qnrB*/*aac(6')-Ib-cr* genotype for which mean inhibition zone diameter was 13 mm compared to 17 mm for 8 isolates with an *oqxAB*/*qnrB* genotype.

### Tetracycline

We tested Kp isolates for tetracycline (n = 57) and tigecycline (n = 48) resistance. While breakpoints for tetracycline are not standardised^31^, the results indicate very high resistance to the antibiotic (Fig. S4). The only five isolates susceptible to tetracycline were collected between years 1980 and 1982, which suggests in the remaining 52 isolates that resistance is acquired rather than intrinsic. Isolates with *tetABD* efflux pumps (n = 31/57) had marginally more resistant profiles. In contrast to tetracycline, most of tigecycline tests inhibition zone diameters were located just below the breakpoint, with only a few isolates with < 10 mm (Fig. S4). Kp does not have an EUCAST zone diameter breakpoint, so we used the *E. coli* 18 mm breakpoint instead. Interestingly, the *tetABD* efflux pumps did not reduce the inhibition zone diameter. Isolates without these efflux pumps were marginally more (mean of 14 mm versus 17 mm), not less, resistant.

### Aminoglycosides

Among isolates tested with gentamicin (n = 349) and amikacin (n = 29), 89% and 55% were resistant, respectively. In those isolates that had no known gentamicin resistance determinants (n = 28), the average inhibition zone diameter was 15 mm versus an established breakpoint of 18 mm (Fig. 4). Two acetyltransferases were present in our isolates ((AAC(6’)-I, n = 283; AAC(3)-II, n = 291), of which AAC(3)-II was a stronger determinant of resistance, with nearly all inhibition zone diameters below 10 mm. AAC(6’)-I reduced the inhibition zone diameter from 15 to 12 mm, and the effect was cumulative with AAC(3)-II. Nucleotidyltransferase ANT(3’’)-I and phosphatases APH(6’)-I and APH(3’)-I, even combined, appear to have a marginal effect on resistance. The 16S rRNA methyltransferase gene *armA* was present in two isolates, and the gene *rmtB* was present in two additional isolates.

### Virulence determinants

Using VFDB, Kleborate and BLAST tools, we examined the dataset for virulence genes^33–35^. The type 1 fimbriae locus, *fimABCDH*, was present in nearly all isolates (501/509, 98%). It was absent in isolates from ST960 (4/4), ST15 (3/161) and ST76 (1/3). Similarly, the type 3 fimbriae locus, *mrkABCDF*, was present in all isolates except for one (ST147). In contrast, iron uptake locus *kfu* was present in 245 (50%) isolates. This locus was perfectly correlated with ST; no ST had simultaneously *kfu* positive and negative isolates. Among the most frequent STs, ST15 (n = 161), ST13 (n = 26) and ST14 (n = 26) isolates had a *kfu* locus, but it was absent in ST147 (n = 36), ST348 (n = 22), and ST307 (n = 20) isolates. There were only a few uncommon virulence genes. For example, we did not find any *rmpA* and *rmpA2* regulators of hypermucoviscosity. Two isolates (Kp4248 and KpV9) had aerobactin siderophores: one with iucA2 (ST3) and the other with iuc3 (ST3027). Both isolates had limited known AMR determinants. ST3 had only *bla*_*SHV-1*_ and ST3027 had APH(6’)-Ia/d, *tet(A)* and *bla*_*SHV-33*_. The salmochelin locus *iroBCDEN* was found only in some ST48 isolates (n = 7/9), all of which carried ESBL *bla*_*CTX-M-15*_. Of these 7 isolates, 6 came from the same Lisbon hospital between years 2005 and 2009. Yersiniabactin was present in 56% of isolates, and was carried most frequently on integrative conjugative elements ICEKp3, ICEKp4 and ICEKp12 at 21%, 15% and 6% of all isolates, respectively. Additional screening against the VFDB virulence database only revealed *astA* and *cseA* genes in one isolate.

## Discussion

In this work we have analysed whole genome sequence data from 509 Kp isolates collected between years 1980 and 2019 from the hospital systems across southern, central, and northern regions of Portugal, as well as from veterinary clinics and a sewage treatment plant in the southern region. Because this is one of the largest in-country collections sequenced, it allowed us to investigate and understand the temporal and spatial genetic diversity of this important pathogen. We observed that 31% of isolates belonged to STs that are considered infrequent in Portugal. If the ST diversity of the dataset was driven by mutations within ST determining genes, we would expect that most samples differ by a single allele, but this was not the case. This observation is further supported by SNP distances between different STs. Instead, the diversity is more likely to be driven by either recombination or coexistence of many strains. The simultaneous presence of so many STs in the country is suggestive of importation and large environmental or human reservoirs of infection. The latter is consistent with high rates of colonisation observed in different countries and settings^36^. The non-human sourced isolates from wastewater and animal settings did not standout in the analysis, which suggests a flow of Kp between humans and environmental reservoirs. However, the limited number of non-human sourced isolates did not allow us to determine the direction of this flow.

Our WGS sequencing analysis revealed insights into AMR genotyping and phenotyping. During the period with best isolate coverage, years 2000 to 2019, the dominant beta-lactam and carbapenem resistance determinants were *bla*_*CTX-M-15*_ (41%) and *bla*_*KPC-3*_ (21%). In isolates from years 1990 to 1999, a majority (11 of 16) had *bla*_*TEM-10*_, which is thought to be the dominant ESBL during that period. Our analysis of plasmids reveals a complex and mosaic distribution across isolates suggestive of active selection between them. The increase in prevalence of *bla*_*KPC-3*_ has been accompanied by a decrease in the frequency of older *bla*_*CTX-M-15*_, which encodes narrower spectrum beta-lactamase. We observed very strong clustering of isolates by their detected plasmid replicons. Plasmid naming nomenclature is based on shared replication mechanisms and incompatibility^37^, so we expected to observe some structure. The replicons themselves revealed a rigid pattern that is clinically relevant due to carriage of *bla*_*KPC-3*_ on two types of plasmids. Isolates with FIA(pBK30683) and FII(pBK30683) replicons were possible sources of *bla*_*KPC-3*_, while isolates with IncN and IncFIB(pKPHS1) were restricted to ST147 types. However, the isolates with *bla*_*KPC-3*_ had an exact same replicon allele present for at least 10 years and formed a very clear cluster of isolates (denoted as replicon cluster 9).

While our AMR test results may suffer from changes in the amount of active compound in test disk assays across the years, we did find interesting and robust results. As expected, we found that *bla*_*KPC-3*_ was the dominant carbapenemase gene^8,12,13^, but we also observed a reduction in carriage of ESBL *bla*_*CTX-M*_ in those isolates that acquired *bla*_*KPC-3.*_ This displacement of *bla*_*CTX-M*_ indicates active selection, and co-carriage of *bla*_*CTX-M*_ and *bla*_*KPC-3*_ was very rare. While AMR genotype was largely consistent with phenotype, we observed that cefoxitin, a retired second-generation cephalosporin shows moderate activity in isolates lacking class C beta-lactamases. We also found that nearly half of the isolates in replicon cluster 9, which had almost universal carriage of *bla*_*KPC-3*_, were susceptible to fluoroquinolones as they lacked *gyrA* mutations or other resistance factors. More generally, such insights may offer opportunities for additional treatment of infections with *bla*_*KPC-3*_ carrying Kp.

Overall, our work has provided temporal and spatial insights into Kp STs and AMR related genes and plasmids circulating in Portugal. We found a large diversity of STs, with ST15 and ST147 being the most frequent (< 40%), but almost one-third of isolates had uncommon types (< 2% frequency). Dominant beta-lactamase genes are changing over time due to changes in drug utilisation and plasmid changes. The *bla*_*OXA-9*_ and *bla*_*TEM-1*_ of the 1980s were displaced by *bla*_*CTX-M-15*_ in 2000’s which in turn were replaced by *bla*_*KPC-3*_. These insights reinforce the need for genomic sequencing and tools to assist surveillance and clinical decision making.

## Methods

### Isolate collection, library preparation and sequencing

The isolates (n = 509) were identified between years 1980 and 2019 from 16 hospitals in Lisbon and its metropolitan area (Southern region), Coimbra (Central region), and Porto and Vila Real (Northern Portugal), except for 9 isolates from Beirolas wastewater (Lisbon) and 41 samples from veterinary clinics (Lisbon) (Table 1). The isolates were cultured as described previously^8^. The set of isolates represent a convenience sample accumulated over 40 years, with the collection site known for the majority (74%). Isolates with known collection site were sourced from blood (32%), urine (31%), rectal screening swabs (10%), pus (7%) and wastewater (7%). Clinical isolates obtained from hospitals were identified at local clinical microbiology laboratories and sent to the Faculty of Pharmacy (University of Lisbon; FFUL) for further phenotypic and genotypic analysis. Given the wide temporal span of the isolates, the initial identification methods that were employed at local laboratories vary across isolates, but were based on biochemical identification methods (e.g., API, Vitek). DNA was extracted from strain cultures grown overnight at 37ºC on Mueller–Hinton Agar. DNA extraction was carried out using the Cetyl trimethylammonium bromide method^38^. Library preparation of the DNA samples was performed using a QIAseq FX DNA library kit, following the manufacturer’s protocol. WGS was performed on Illumina HiSeq (paired end 150 bp) through The Applied Genomics Centre (London School of Hygiene and Tropical Medicine)^39^. Only those isolates which Kleborate software (v 2.1.0)^40^ identified as Kp were used for further analysis.

### Antimicrobial susceptibility testing

Antimicrobial susceptibility testing at FFUL was carried out using the Kirby-Bauer disk diffusion method as per the European Committee on Antimicrobial Susceptibility Testing (EUCAST) guidelines for performance and interpretation of antimicrobial susceptibility testing (v11.0, 2021)^31^. AMR testing was performed over multiple years, and therefore the concentration of active compounds in disks used might vary between isolates. The AMR testing was not performed for 121 isolates, and not all isolates were tested for the same antimicrobials. The Pearson correlation coefficient was used to assess correlations between disk inhibition zone diameters for different antimicrobials.

### Genome assembly, annotation, and genotyping

Raw Illumina reads were assembled using Unicycler software (v0.4.8)^41^, with assembly fragmentation and completeness assessed against 440 core genes of enterobacterales (enterobacterales_odb9) using Busco software (v4)^42^. The assessed quality of assemblies was high (median N50: 284Kbp) with all but one having complete single copies of > 97% genes in the Busco reference set. Assemblies were annotated using Prokka software (v 1.14.6)^43^, combined with the *Klebsiella* specific reference genes set^44^. O and K antigen serotypes*,* genomic AMR, virulence (e.g., ICEKps), and sequence type (ST) profiles were analysed and inferred in silico using Kleborate^40^ and AMRFinder (v3.8.4)^45^ software with associated databases (accessed October 2020). We have also used Abricate software (v1.0.1)^46^ with the virulence factor database VFDB (accessed March 2021) to find additional virulence genes. Plasmid detection and classification was performed using Plasmidfinder software (v2.1.1)^43^.

### Phylogenetic analysis

The recombination and horizontal gene transfer within Kp can complicate phylogenetic analysis, and a widely used Kp MLST scheme^22^ does not allow for a high-resolution phylogeny. We have observed that regions linked to transposons and other genes relating to mobile genetic elements tended to produce high number of SNPs, which would bias phylogenetic reconstruction. For this reason, instead of performing phylogenetic reconstruction using SNPs called against a reference genome, we used a reference-free method. We focused on a subset of core genes defined by two conditions. First, in all isolates, the gene coding sequence length is within 1% of median gene length across isolates. Second, the genes have pairwise identity of > 99%. Based on these criteria, out of ~ 5,000 genes in Kp isolates, 1424, 2212, 2802, and 3170 genes were present in 100%, 99%, 95% and 90% of all 509 isolates. We used Shannon’s entropy to identify a 100 (from the 1424) genes with the most diverse nucleotide sequences. These 100 genes were aligned used MAFFT (v7.467)^48^ and the resulting alignments used to construct phylogenetic trees for the entire isolate set (n = 509). The trees were reconstructed using IQTREE (v2.0.3)^49^ with 1000 bootstrap replicates for each tree. The scripts can be found at https://github.com/AntonS-bio/entropy. For each ST, the gene selection was performed separately. The location of each chromosomal gene on the Kp reference genome (NC_016845.1) (Fig. S1) was generated with BRIG software (v 0.95)^50^. Phylogenetic trees were visualised in ITOL and are available (https://itol.embl.de/shared/Zp28yLE9IuWB).

### Statistical analysis

Statistical analysis was performed using R software (v4.0.3)^51^. Additional analysis was performed in Python (v3.6). For detection of replicon clusters, we created a presence/absence matrix with one row per isolate and one column per each unique replicon sequence. We performed dimensional reduction on this matrix using the Uniform Manifold Approximation and Projection (UMAP) algorithm^29^ implemented in the R uwot package. We used both hamming and jaccard distance measures and a broad range of parameters to establish the robustness of our results. The cluster detection was performed using the DBSCAN algorithm implemented in R^52^. Analysis scripts are available on https://github.com/AntonS-bio.

### Consent for publication

All authors have consented to the publication of this manuscript.

## Supplementary Information

Supplementary Information 1.

Supplementary Information 2.

Supplementary Information 3.

## Data Availability

All sequencing data is available from European Nucleotide Archive project PRJEB47288. All AMR testing data is available in PATRIC database. Analysis scripts are available at https://github.com/AntonS-bio. High resolution phylogenetic trees are available at https://itol.embl.de/shared/Zp28yLE9IuWB.
