# Supplementary material for: Genomic epidemiological analysis of Klebsiella pneumoniae from Portuguese hospitals reveals insights into circulating antimicrobial resistance
Source: Sci Rep. 2022 Aug 13;12:13791. doi: 10.1038/s41598-022-17996-1 (PMC9375070; doi:10.1038/s41598-022-17996-1)
Supplement: Supplementary file 3 — Supplementary Information 3. [file 41598_2022_17996_MOESM3_ESM.docx]

**Supplementary Tables**

**Table S1.** Resistance phenotypes of isolates undergoing drug susceptibility tests (DSTs)

| **Antimicrobial**  **Family** | **Name** | **Abbrev.** | **DST**  **N** | **DST Resistance N** | **DST Resistance**  **%** |
| --- | --- | --- | --- | --- | --- |
| Aminoglycosides | gentamicin | gm | 356 | 313 | 88 |
|  | amikacin | ank | 29 | 16 | 55 |
| Carbapenems | imipenem | ipm | 296 | 68 | 23 |
|  | meropenem | mem | 45 | 32 | 71 |
|  | doripenem | dor | 12 | 12 | 100 |
| Cephalosporins 2nd | cefoxitin | fox | 366 | 157 | 43 |
| Cephalosporins 2nd | cefuroxime | cxm | 21 | 14 | 67 |
| Cephalosporins 3rd | cefotaxime | ctx | 362 | 279 | 77 |
| Cephalosporins 3rd | ceftazidime | caz | 357 | 303 | 85 |
| Cephalosporins 3rd | ceftazidime-avibactam | cazAvb | 45 | 1 | 2 |
| Cephalosporins 3rd | ceftriaxone | cro | 28 | 22 | 79 |
| Cephalosporins 4th | cefepime | fep | 39 | 35 | 90 |
| Fluoroquinolones | ciprofloxacin | cip | 344 | 258 | 75 |
|  | levofloxacin | lev | 35 | 20 | 57 |
|  | norfloxacin | nor | 23 | 13 | 57 |
| Miscellaneous agents | fosfomycin | fos | 27 | 18 | 67 |
| monobactam | aztreonam | atm | 86 | 77 | 90 |
| Penicillins | amoxicillin-clavulanic acid | amc | 311 | 292 | 94 |
|  | amoxicillin | amx | 56 | 56 | 100 |
|  | piperacillin | pip | 45 | 45 | 100 |
|  | ticarcillin-clavulanic acid | tcc | 34 | 34 | 100 |
|  | ticarcillin | tic | 34 | 34 | 100 |
| Tetracyclines | tigecycline | tig | 48 | 40 | 83 |

**Supplementary Figures**

**
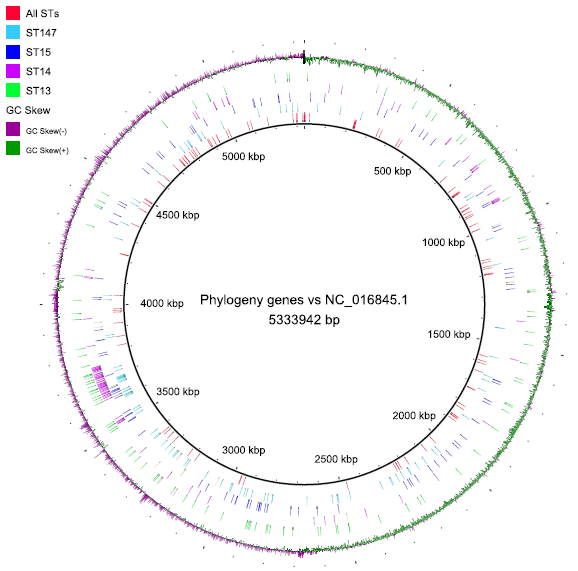
**

**Figure S1.** Location of Kp genes used for phylogenetic reconstructions in **Figure 1**, with their presence across sequence types (STs).

**Figure S2.** Multi-locus sequence typing (MLST) allele distance between unique sequence types.

**
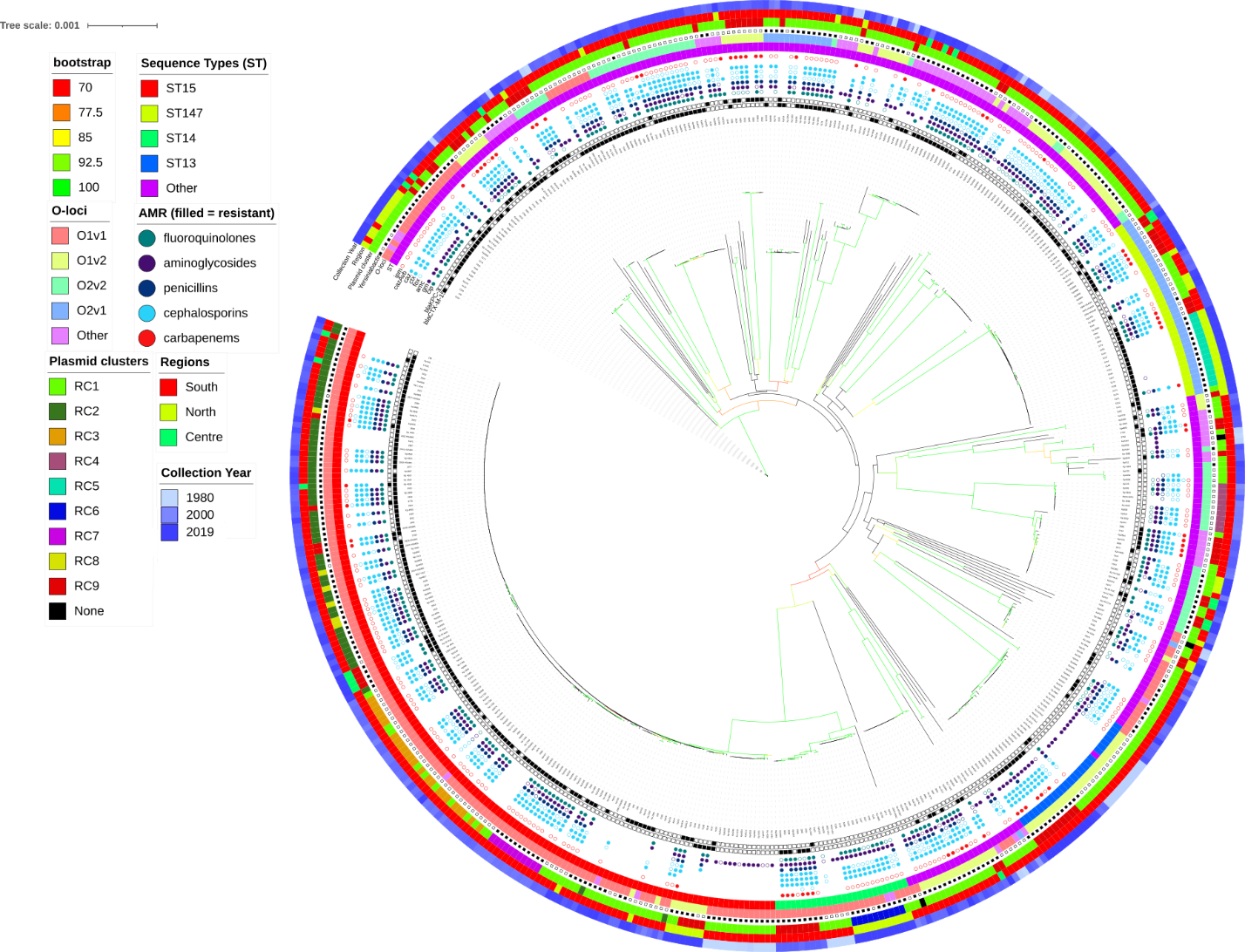
**

**Figure S3.** Maximum likelihood phylogenetic tree for all isolates (n=509) using core genes; O-loci are inferred O serotypes; ST sequence types; RC replicon cluster; boot = bootstrap support %; AMR antimicrobial resistance.


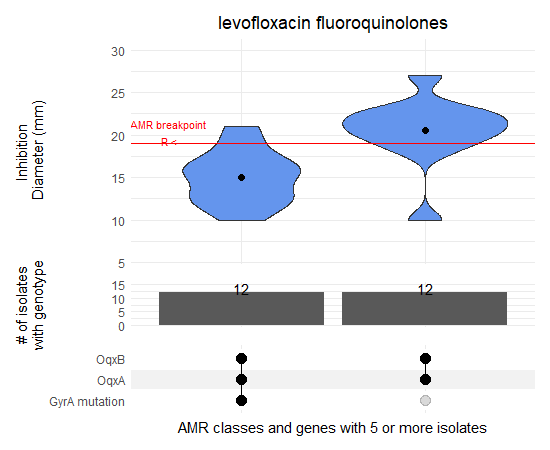


**(B)**


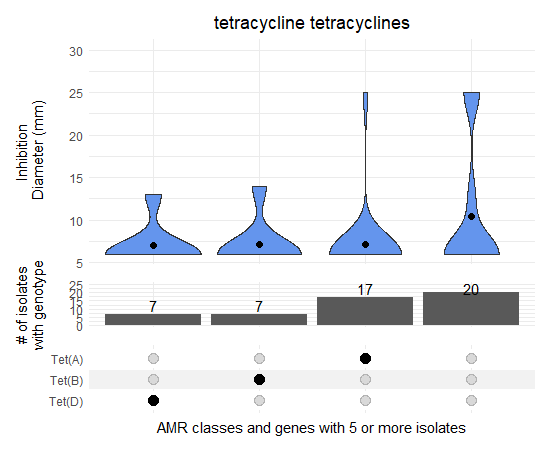


**(C)**


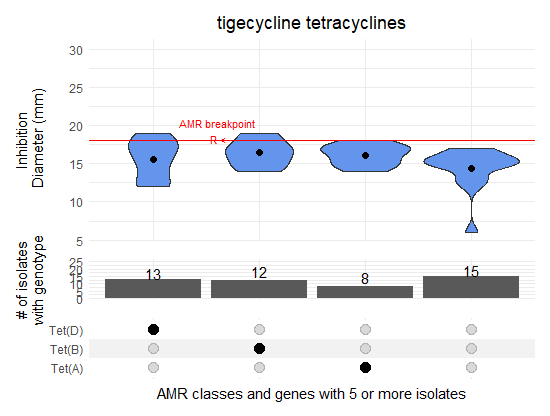


**Figure S4.** Inhibition zone diameters for selected antimicrobials **(A**) Levofloxacin; **(B)** Tetracycline; **(C)** Tigecycline. Note, EUCAST does not have a Kp breakpoint for tetracycline. For tigecycline, EUCAST v11.0 does not define a Kp breakpoint, so *E. coli* breakpoint is used instead.

**(A)**

**
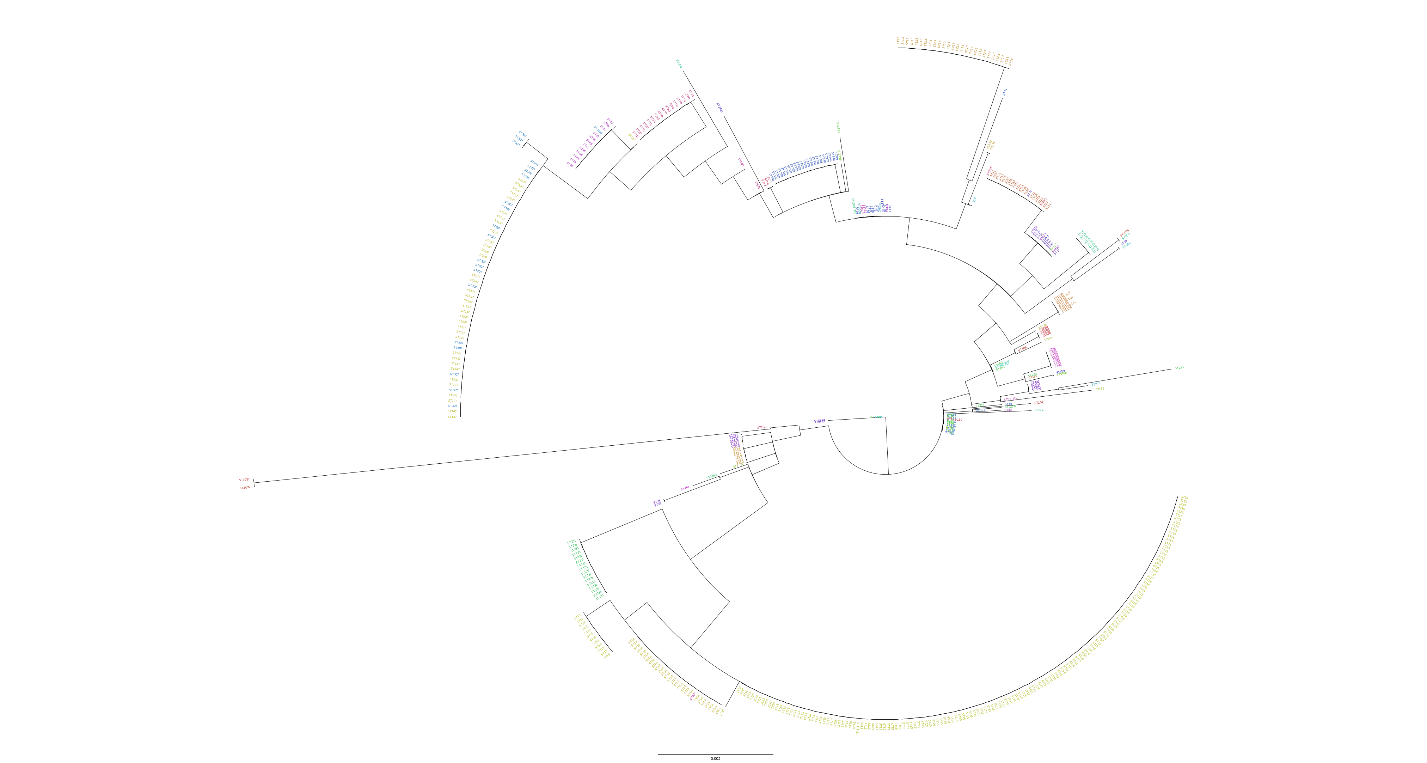
**

**(B)**

**
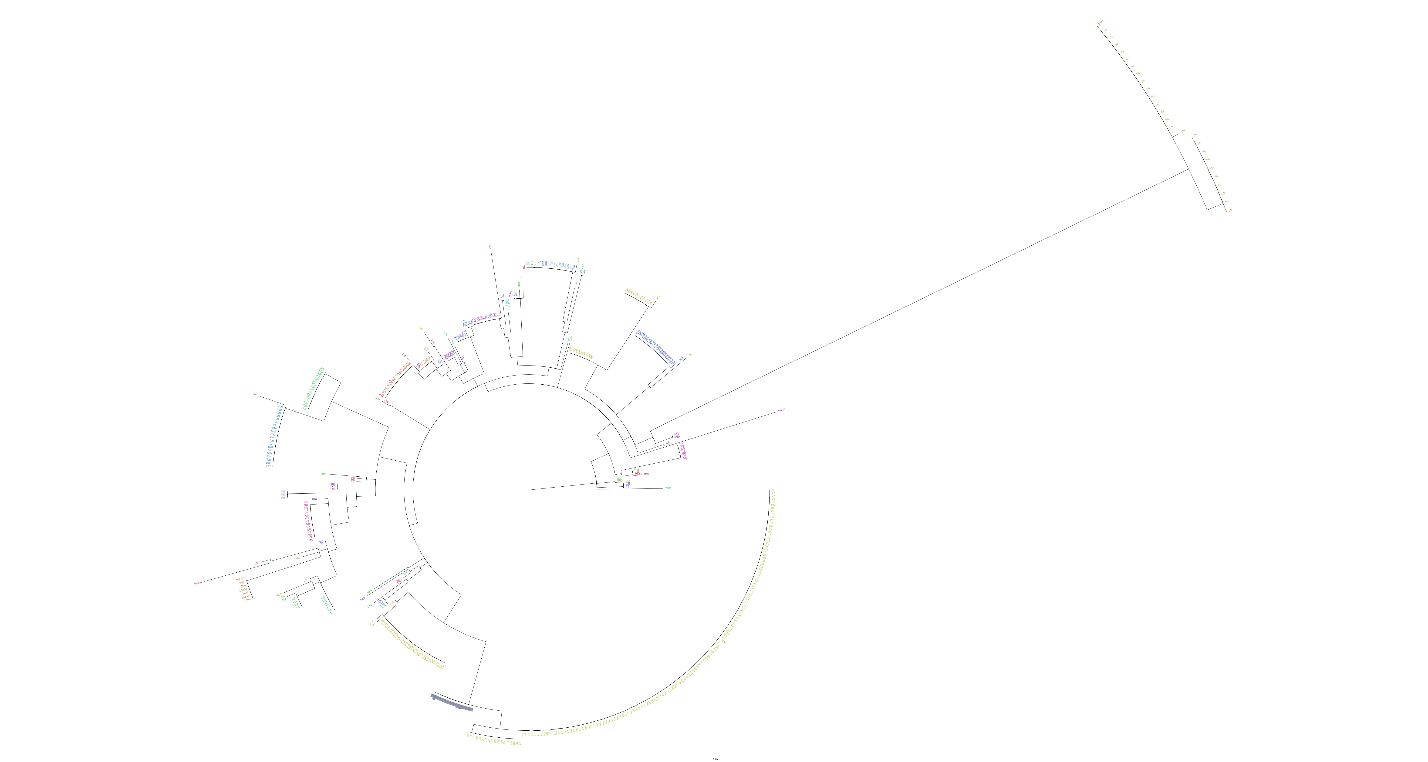
**

**Figure S5.** Maximum likelihood phylogenetic tree for all isolates (n=509) using the: **(A)** *parC* gene; (**B)** *gyrA* gene.
